# New York Academy of Medicine

**Published:** 1864-05

**Authors:** James Anderson

**Affiliations:** President, in the Chair


### NEW YORK ACADEMY OF MEDICINE.

STATED MEETING, April 6, 1864.

DR. JAMES ANDERSON, PRESIDENT, IN THE CHAIR.

### CEREBRO-SPINAL MENINGITIS.

Dr. DETMOLD read an account without comment, of a case of cerebro-spinal meningitis, called also spotted fever, occurring under his observation, after which Dr. W. H. Draper read a portion of an interesting paper which he is preparing on the same disease. His paper is founded upon a somewhat lengthy and careful observation, principally in the neighborhood of Carbondale, Pa., where the disease has been raging to a fearful extent. As the paper is not yet completed, no analysis of it can be given at present. Dr. Skiven, of Long Branch, N. J., being present, by invitation, proceeded to address the Academy at some length, giving a minute description of the disease as it appears in his locality. The neighborhood where the disease has occurred with the greatest degree of severity is but a short distance from the sea-shore. The climate during the winter has been mild, there having been no storm from the ocean until some time in the month of March. The ground is generally dry, there being no marshes or swamps that are not covered by the tide. The spring and well water is more or less impregnated with chloride of sodium and the salts of iron. The mode of living is varied, there being no uniform system of diet, either in meats, drinks, or vegetables. The population is composed of different classes, from the poor laborer to those in easy circumstances; all seem happy and contented, and enjoy life much. From these facts there seems to be no known cause from soil, climate, or mode of living, by which we can account for the appearance of the disease. It was observed that as soon as this disease appeared all other diseases disappeared, or seemed to be swallowed up in this. During last winter, measles prevailed to an extent and degree of severity hitherto unknown. It attacked persons of all ages, from the young child to the old man of seventy. In past years this has invariably been followed by scarlatina; but this year it was not so; upon the disappearance of measles from the neighborhood, cases of cerebro-spinal meningitis began to appear. The first symptom of the disease has generally been a severe pain in the knee, sometimes in the hands, and attended by what the patient describes as "pain in the bones," extending along the direction of the limb. After a short time, or in some instances a day or two, the pain leaves the limbs and attacks the head, from which it extends to the back of the neck, and continues down as far as the lumbar region. This, like the pain in the limbs, is aggravated more by motion than pressure; indeed, every attempt at motion is attended by the most intense pain. If the patient can be seen at the commencement of this stage of the disease, he is bled freely from the arm, leeches applied to the temples and mastoid processes, cups to the back of the neck and along the spine, followed by mustard applications and the administration of a brisk cathartic. Before Dr. S. commenced this treatmen, he lost a majority of his patients; but since he began to bled early, a much greater proportion recover. Later, when stimulants seem to be indicated, he has found equal portions of capsicum and camphor in powder, to be attended with a better effect than any of the diffusible stimulants. The prejudice of the inhabitants is against autopsies, consequently the advantages for studying the pathology of the disease are limited. Dr. S., however, exhibited some specimens, consisting of a portion of the dura mater, one of the stomach, and one of the bladder; these all appeared to be in a very high state of congestion. The spots, from which the disease is sometimes called "spotted fever," Dr. S. regards as accidental, being simply spots of ecchymosis caused by rupture of some of the small vessels during the stage of reaction. It was announced that this subject would be continued at the next meeting, after which the Academy adjourned.

